# Management of wound dehiscence with negative pressure in diabetic patients undergoing pulmonary resection for lung cancer

**DOI:** 10.3389/fsurg.2026.1849452

**Published:** 2026-08-06

**Authors:** Annalisa Carlucci, Roberto Cascone, Domenico Caporale, Gabriella Giudice, Cosimo Lequaglie

**Affiliations:** Thoracic Surgery Unit, Centro di Riferimento Oncologico Della Basilicata (IRCCS-CROB), Rionero in Vulture, PZ, Italy

**Keywords:** diabetes mellitus, lung cancer, oncology, thoracotomy, wound

## Abstract

**Background:**

Thoracotomy wound dehiscence, with or without infection, remains a challenging postoperative complication associated with delayed recovery and increased healthcare burden. Diabetes mellitus is a well-recognized risk factor for impaired wound healing. The aim of this study was to evaluate the role of a portable incisional negative-pressure wound therapy (iNPWT) device (PICO™) in the management of thoracotomy wound dehiscence in diabetic patients undergoing pulmonary resection for lung cancer.

**Methods:**

This small retrospective comparative case series included 19 consecutive diabetic patients who developed thoracotomy wound dehiscence after pulmonary resection for lung cancer between February 2006 and June 2018. Following surgical wound revision, debridement, and complete wound re-closure, 9 patients received iNPWT with the PICO™ system (Group A), while 10 patients received conventional dressing (Group B). Continuous variables were expressed as median and interquartile range (IQR) and compared using the Mann–Whitney U test. Categorical variables were analyzed using Fisher's exact test. Statistical significance was set at *p* < 0.05.

**Results:**

Baseline demographic and clinical characteristics were comparable between groups. Patients treated with the PICO™ system showed a significantly shorter time to complete wound healing compared with those receiving conventional dressing [10 days [8.25–12] vs. 13 days [12–14], *p* = 0.0238]. Complete wound healing was achieved in all patients. No significant differences in pain scores were observed between groups.

**Conclusions:**

In this small retrospective comparative case series, the use of the PICO™ system was associated with a shorter time to complete wound healing in diabetic patients with thoracotomy wound dehiscence. Given the retrospective design and limited sample size, these findings should be interpreted with caution. Larger prospective studies are warranted to confirm the potential role of incisional negative-pressure wound therapy in this clinical setting.

## 1 Introduction

In surgery, wound dehiscence is a frequent complication that can have a major impact on surgical outcome. Even a small opening is a gateway to infection, and thus it should be treated early. The incidence of dehiscence depends on the patient's condition, type of surgery and has a potential negative impact on the quality of life and costs. Many risk factors influence the effectiveness of wound healing, such as the time of intervention, obesity, revision surgery, age, diabetes, tobacco abuse. Randomized controlled trials, retrospective studies and case series show that negative pressure wound therapy (NPWT) promote the healing of dehiscence of surgical wounds with consequent reduction of mortality and morbidity in different surgical specialities (e.g., orthopaedic, general surgery, head and neck surgery, cardiothoracic, and plastic surgery) (1–4). However, no data are reported so far regarding the use of NPWT for treating wound surgical infection after thoracic surgery procedures in patients with diabetes mellitus (DM).

DM is an endocrine pathology that can cause alterations at the vascular level, with both macrovascular and microvascular damage. It is precisely this latter mechanism that contributes to counteracting wound healing since the alteration of the microcirculation prevents correct tissue nutrition, inflammatory response and removal of waste products.

Although wound dehiscence is a known complication in patients undergoing surgery, the presence of DM increases its incidence with a consequent prolongation of hospitalization and related events (i.e., higher incidence of nosocomial infections, increased hospital costs, etc.).

The aim of this paper was to investigate the effectiveness of a portable NPWT device compared to traditional treatment for management of wound dehiscence after thoracic surgery procedures in diabetic patients (5, 6).

## 2 Materials and methods

### 2.1 Study population

This study was designed as a small retrospective comparative case series evaluating diabetic patients who developed thoracotomy wound dehiscence and were treated with either incisional negative-pressure wound therapy (PICO) or conventional dressing.

In these case series we included 19 consecutive patients who underwent pulmonary resection for lung cancer at our center from February 2006 to June 2018 and who developed wound dehiscence as a postoperative complication. For the purposes of this study, wound dehiscence was defined as any postoperative separation of a thoracotomy incision involving the skin, subcutaneous tissue, and/or muscular layer after primary wound closure, with or without evidence of local infection. Both infected and non-infected dehiscence were included. Patients without a history of diabetes mellitus and cases associated with tissue loss preventing complete wound-edge re-approximation were excluded from the study. No cases of full-thickness thoracotomy dehiscence involving the chest wall or requiring complex reconstructive procedures were included. An additional inclusion criterion was the presence of non-insulin-dependent diabetes mellitus treated with oral hypoglycemic agents. The characteristics of the study population are summarized in Table 1. In 9 patients we used the PICO system to manage the wound dehiscence (Group A) while the remaining 10 patients were treated with only traditional therapy, without this device (Group B). Allocation to the PICO treatment group was based exclusively on device availability at the time of diagnosis of wound dehiscence. No predefined clinical, demographic, or wound-related criteria were used to assign patients to either treatment group. Demographics, length of thoracotomy, post-operative pain, and effective healing time were recorded for all patients and the data collected from the two groups were subsequently compared. No patients included in the study had chronic renal disease or had received chemotherapy or radiotherapy before the occurrence of wound dehiscence. Complete wound healing was defined as the absence of wound separation with complete approximation of the wound edges, allowing safe removal of skin sutures. Wound healing was assessed by clinical examination during postoperative follow-up. The time to complete wound healing was calculated from wound revision surgery to the first clinical assessment documenting complete wound closure and suture removal. All participants signed a written informed consent for the treatment and for collecting their data for scientific purposes.

**Table 1:** Data of study population.

Variable	Group A (*n* = 9)	Group B (*n* = 10)	*p-value*	Effect size
Age (years)	67 (60.5–72.5)	66.5 (60–72)	1.000	rbb = 0.00
Sex (M)	5 (55.56%)	5 (50%)	1.000	OR 1.25 (95% CI 0.21–7.62)
HbA1c	56 (55–57)	55.5 (53–57)	0.8371	rbb = 0.47
Body mass index	22 (21–24.25)	25.5 (23–26)	0.0854	rbb = 0.467
Serum albumin (g/dL)	3.8 (3.62–4.12)	3.8 (3.5–4)	0.7133	rbb = 0.10
Smoking status	7 (77.78%)	7 (70%)	1.000	OR 1.50 (95% CI 0.18–12.28)
Operative time (min)	120 (110–130)	120 (110–120)	0.7395	rbb = 0.09
Lenght of stay (days)	7 (6–8.25)	16 (15–17)	0.0002	rbb = 1.00
Cardiac comorbidities	3 (33.3%)	2 (20%)	0. 6284	OR 2.00 (95% CI 0.24–16.97)
COPD	2 (22.2%)	4 (40%)	0. 6284	OR 0.43 (95% CI 0.05–3.76)
Thoracotomy length (cm)	13 (11.5–14.25)	13 (12–15)	0.7114	rbb = 0.10
Complete wound healing (days)	10 (8.25–12)	13 (12–14)	0.0238	rbb = 0.61
Pain/discomfort (VAS scale)	2 (0.75–2.25)	2 (1–3)	0.5063	rbb = 0.18

### 2.2 Diabetes mellitus and wound dehiscence management

During the study period, the institutional protocols for surgical management of wound dehiscence, glycemic control in diabetic patients, and antibiotic therapy remained substantially unchanged.

In 15 patients, the glycated hemoglobin (HbA1c) level was above 53 mmol/mol, indicating poorly controlled diabetes at the time of surgery. During hospitalization, oral hypoglycemic therapy was replaced with insulin therapy based on blood glucose testing, achieving blood glucose levels within the normal range. No other complications related to the presence of diabetes mellitus were reported in the patients analyzed. Patients were splitted in two groups according to whether they were treated with PICO device (Group A) or with traditional therapy (Group B). Regardless of the group they belonged to, all patients underwent a reoperation in the operating room.

A wound swab was always primarily performed to detect the possible presence of infection. The infectious agent at the wound site was identified in 9 patients (5 in Group A and 4 in Group B), as shown in Table 2. In these patients, systemic antibiotic therapy was administered based on the results of the antibiogram.

**Table 2:** Here are summarized the microbiological findings of culture-positive wounds and is presented for descriptive purposes only. These data were not included in the comparative statistical analysis because wound cultures were not positive in all patients.

Patient no.	Group	Culture test
1	A	*Escherichia Coli*
2	A	*Enterobacter*
3	A	*Escherichia Coli*
4	A	*Staphylococcus Aureus*
5	A	*Pseudomonas Aeruginosa*
6	B	*Klebsiella Pneumonia*
7	B	*Klebsiella Pneumonia*
8	B	*Staphylococcus Aureus*
9	B	*Staphylococcus Aureus*

In all cases, a revision of the thoracotomy was performed from the skin level down to the muscle layer, with thorough debridement and removal of any necrotic tissue. The surgical procedure was completed with a new closure of the thoracotomy. No metal clips were applied to the skin; instead, a resorbable monofilament suture was used.

At the end of the debridement, the PICO device was applied to the patients of group A while the patients of group B received a standard dressing. Specifically, the PICO device was applied immediately after surgical wound revision and complete re-closure of the thoracotomy incision whenever full approximation of the wound edges was achievable. Therefore, PICO was used as incisional negative-pressure wound therapy (iNPWT) over a closed surgical incision and replaced the conventional postoperative dressing used in the control group.

**Figure 1:**
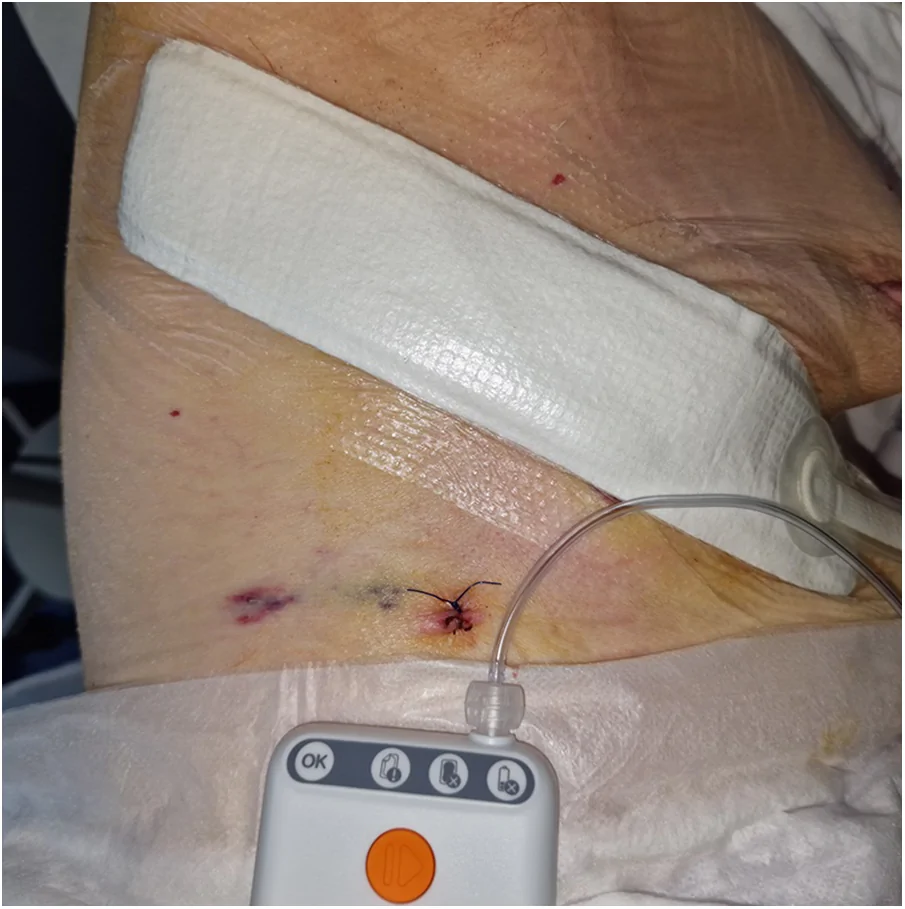
Application of the PICO™ device over a closed thoracotomy incision following surgical wound revision, debridement, and complete re-closure of postoperative wound dehiscence.

**Figure 2:**
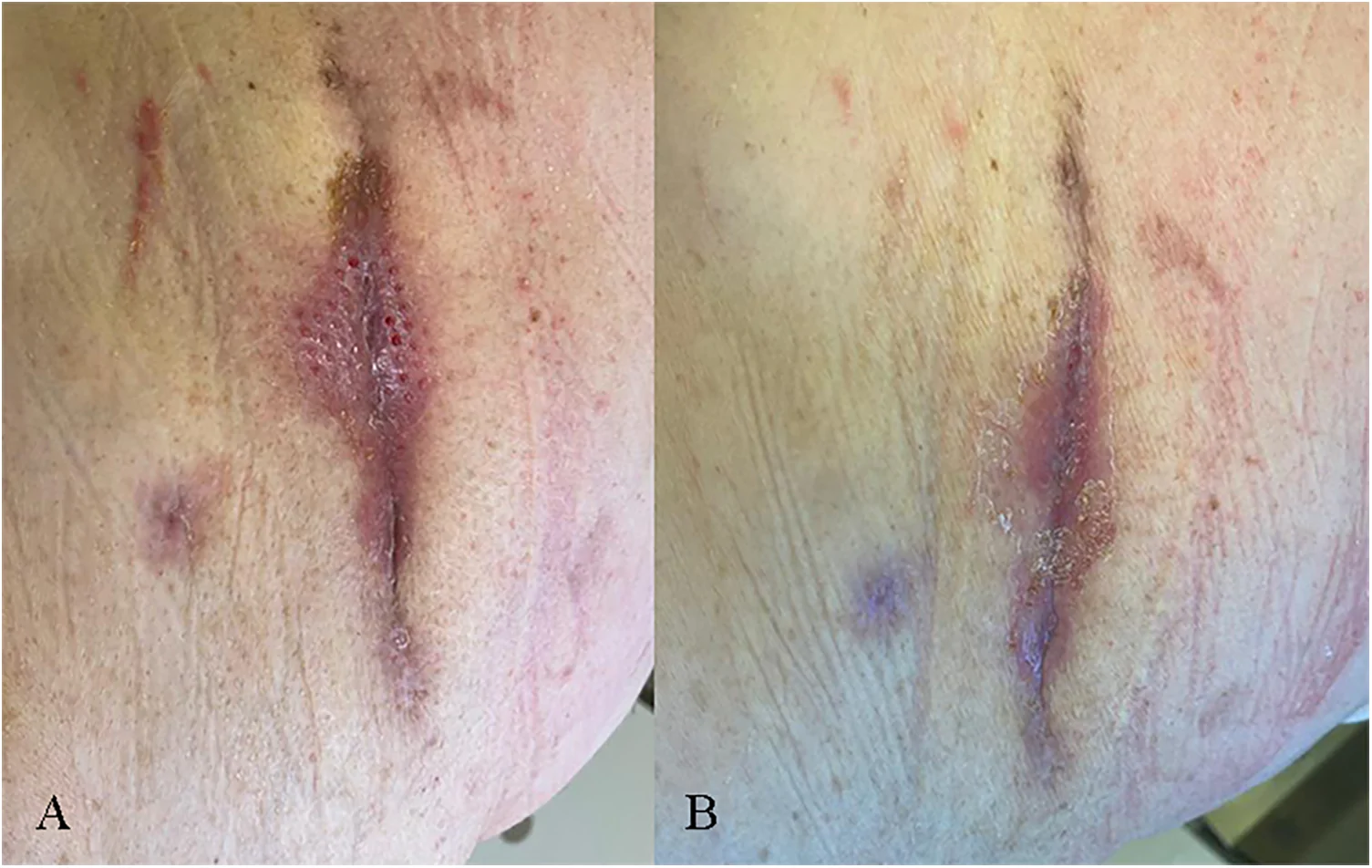
Representative clinical outcome following treatment with the PICO™ system. **(A)** Thoracotomy incision 7 days after application of incisional negative-pressure wound therapy (iNPWT). **(B)** Complete wound healing 10 days after treatment, with complete wound-edge approximation and readiness for suture removal.

PICO device (Figure 1) is a portable device that uses a negative pressure and a specific dressing that help to maintain the incision edges together, redistributes lateral tension, reduces edema, stimulates perfusion, enhances the development of granulation tissue and protects the site from external infections (Figure 2). When the suction pump of this device is turned on, air is pulled out of the dressing creating negative pressure of −80 mmHg and drawing excess fluid from the wound. Thanks to the small size compared to the classic VAC therapy devices, it was possible to discharge patients early as it is easily manageable at home. Patients with PICO were re-evaluated every three days in the outpatient department, replacing the dressing when required.

Since the battery of the device has a duration of 7 days, where necessary it has been replaced at its natural expiry.

To verify whether the application of continuous negative pressure could cause significant pain or discomfort, the 19 patients were asked to quantify with an analog scale from 0 to 10, where 0 represents the absence of pain and 10 the maximum perceptible pain.

Moreover, at outpatient follow-up, patients treated with the PICO device were asked whether they were able to gradually resume their usual daily activities and maintain personal autonomy during treatment. This patient-reported assessment was recorded as a dichotomous variable (Yes/No) and was used as a simple indicator of quality of life during home management.

### 2.3 Statistical analysis

Continuous variables were expressed as median and interquartile range (IQR), whereas categorical variables were reported as absolute frequencies and percentages. Comparisons between groups were performed using the Mann–Whitney U test for continuous variables and Fisher's exact test for categorical variables. Effect size for continuous variables was estimated using rank-biserial correlation, whereas odds ratios (ORs) with 95% confidence intervals (95% CIs) were calculated for categorical variables. All tests were two-sided, and a *p*-value <0.05 was considered statistically significant. MedCalc Statistical Software version 14.8.1 (MedCalc Software bvba, Ostend, Belgium) was used for analysis.

## 3 Results

The data are reported in Table 1. The two groups were well matched, and no statistical differences were found regarding the age, the gender, the co-morbidities, and the length of thoracotomy. It was observed that the time required for complete wound healing in the PICO group was significantly shorter than in patients with standard dressing [10 days (8.25–12) in Group A vs. 13 days (12–14) in Group B - *p* = 0.0238 – rbb = 0.61].

Pain reported by PICO patients, in contrast, did not appear to be statistically significant compared to patients who did not have negative wound pressure applied [2 (0.75–2.25) in Group A vs. 2 (1–3) in Group B – *p* = 0.5063 - rbb = 0.18].

At follow-up evaluation and device removal, all patients treated with the PICO system reported preservation of personal autonomy and gradual resumption of daily activities, suggesting good tolerability of home-based wound management.

## 4 Discussion

Several patient-related factors, including diabetes, obesity, peripheral vascular disease, renal failure, malnutrition, chronic obstructive pulmonary disease, prolonged mechanical ventilation, tobacco use, radiotherapy, chemotherapy, and immunosuppressive treatment, have been associated with impaired wound healing and an increased risk of surgical wound complications (7–10).

DM is a disease known to be associated with worse outcomes in lung cancer patients undergoing surgery. When preoperative DM is not well controlled, this condition predisposes to reduced postoperative survival rates. Furthermore, it also has effects on short-term outcome, such as the appearance of wound dehiscence (11–13).

Occurrence of wound dehiscence is well described as complication after thoracic procedures as well as with surgical procedures of other specialty, such as hip and knee arthroplasty lower extremities by-pass, abdominal laparotomy (14–16).

No cases are described in literature about the use of a NPWT in thoracic wounds in diabetic patients. In our study we used a portable suction device, the PICO system, in all high-risk thoracic wound dehiscence to avoid all the complications related with standard VAC therapy.

Negative pressure wound therapy has been reported to promote wound healing and reduce bacterial burden through several biological mechanisms. This technique avoids triggering the inflammation processes that would cause the efflux of fluid into the perivascular space, improving interstitial diffusion of oxygen to cells, and removes deleterious enzymes from the wound such as collagenases and matrix metalloproteinases (MMPs) and other proteases related to inflammatory cells, as well as bacterially derived proteases. By removing the wound fluid and bacteria that inhibit wound healing, PICO modifies the wound microenvironment toward one more conducive to healing. In addition, the cyclic compression and relaxation of the wound tissue likely stimulate mechanotransductive pathways: that results in increased growth factor release, matrix production, and cellular proliferation (17, 18). Our findings are consistent with the existing literature, demonstrating a reduction in wound-healing time. However, it should be emphasized that although patients treated with the PICO system experienced a statistically significant reduction in time to complete wound healing, the absolute difference between groups was modest. Therefore, the clinical significance of this finding should be interpreted with caution, particularly considering the retrospective nature of the study and the limited sample size. Nevertheless, the large effect size observed (rrb = 0.61) suggests a potentially meaningful benefit that warrants further investigation in larger prospective studies. The amount of pressure applied to the surgical site is extremely important. The use of NPWT was described by Tang et al. (19) for the treatment of sternotomy dehiscence after cardiac surgery. In their case series, V.A.C.® therapy was used with a pressure of −125 mmHg applied intermittently, with a healing time of 27 days. In our study, we applied a negative pressure of −80 mmHg in all patients without increasing this value to prevent capillary flow occlusion. The latter event could in fact cause a diffuse lymphatic leakage followed by partial necrosis of the wound, compromising the success of the procedure. Furthermore, due to the low negative pressure values, we chose to apply constant rather than intermittent aspiration, obtaining results in terms of healing time in line with those of the reported study.

NPWT can be a cause of pain or discomfort in patients, especially in the possibility of home management (20, 21) In addition to the pain, which in some cases affects the patient's quality of life, there is also the social discomfort related to the visibility of the NPWT device, such as the VAC®, which limits the subject in daily activities. From the data collected in our study, the pain perceived by the patients at the time of discharge with the portable device was not greater than that of the patients who only received the standard treatment. In our cohort, pain scores were comparable between groups, suggesting that home management with the PICO system was generally well tolerated. Furthermore, the small size of the device we use allows it to be easily concealed, reducing the social discomfort generated by showing a medical device to other people. Patients were discharged home with the portable device *in situ* and followed in the outpatient setting. During follow-up, all patients reported preservation of personal autonomy and gradual resumption of daily activities.

A shorter hospitalization may potentially be associated with reduced healthcare costs, improved patient experience, and a lower risk of hospital-acquired complications. However, these outcomes were not specifically assessed in the present study.

Complications reported with conventional NPWT, including pain, protein loss, and bleeding (22), were not observed in our cohort treated with the PICO system. Nevertheless, if such adverse events occur, treatment should be promptly discontinued and appropriate corrective measures undertaken.

## 5 Conclusions

In this small retrospective comparative case series, the use of the PICO system was associated with a shorter time to complete wound healing compared with conventional dressing in diabetic patients with thoracotomy wound dehiscence. The main limitations of our study include its retrospective nature and the small sample size. In addition, the long study period represents an additional potential source of temporal bias, as well as the lack of standardized baseline images of wound dehiscence. Nevertheless, the institutional protocols for wound management, glycemic control in diabetic patients, and antibiotic therapy remained substantially unchanged throughout the study period, potentially limiting the impact of this bias.

Overall, the results suggest that incisional negative-pressure wound therapy may represent a useful option for the management of selected diabetic patients with thoracotomy wound dehiscence, particularly in the outpatient setting. Larger prospective and multicenter studies are needed to confirm these preliminary findings and to better define the role of PICO therapy in this clinical setting.

## Data Availability

The raw data supporting the conclusions of this article will be made available by the authors, without undue reservation.
